# Heart-reactive antibody levels differentially associate with ventricular function in hereditary cardiomyopathies

**DOI:** 10.1186/s12872-026-06808-3

**Published:** 2026-10-22

**Authors:** Dennis Schiffer, Boshra Afshar, Daria Grosser, Juliana Pet, Lena Schmidbauer, Maximilian Bauser, Gustavo Ramos, Valerie Boivin-Jahns, Roland Jahns, Stefan Frantz, Peter Heuschmann, Stefan Störk, Brenda Gerull, Niklas Beyersdorf, Caroline Morbach

**Affiliations:** 1 Department of Clinical Research & Epidemiology, Comprehensive Heart Failure Center (CHFC), University Hospital Würzburg, Würzburg, Germany; 2 Department of Medicine I, University Hospital Würzburg, Würzburg, Germany; 3 Institute for Virology and Immunobiology, University of Würzburg, Würzburg, Germany; 4 Institute for Clinical Epidemiology and Biometry, University of Würzburg, Würzburg, Germany; 5 Institute for Pharmacology and Toxicology, University of Würzburg, Würzburg, Germany; 6 Interdisciplinary Bank of Data and Biomaterials (ibdw), University Hospital Würzburg, Würzburg, Germany; 7 Department of Cardiovascular Genetics, Comprehensive Heart Failure Center (CHFC), University Hospital Würzburg, Würzburg, Germany

**Keywords:** Genetic cardiomyopathy, Heart-reactive antibodies, Myosin heavy chain, Tropomyosin, Autoimmunity

## Abstract

**Background:**

gCMP are a major cause of cardiovascular morbidity and mortality. While agonistic anti-β1-adrenergic receptor antibodies (anti-β1AR-EC_II_) have a recognized pathogenic role in idiopathic dilated cardiomyopathy (DCM), the relevance of HRA in gCMP remains unclear.

**Methods:**

One hundred gCMP patients with confirmed genetic variants in cardiomyopathy genes were compared with 100 age- and sex-matched healthy individuals. Antibody reactivities were quantified using bead-based flow cytometric assays against cardiac antigens: The second extracellular loop of the β1 adrenergic receptor (β1AR-EC_II_), Troponin I3 (TPI3), Tropomyosin 1 (TPM1), Myosin heavy chain 6 and 7 (MYH6, MYH7) and Myosin light chain 7 (MYL7).

**Results:**

gCMP patients showed higher anti-TPI3 and lower anti-TPM1 antibody levels compared to controls indicating increased induction of the former and loss of the latter in gCMP patients. Age and sex influenced HRA patterns in both groups. Anti-MYH6 and anti-TPM1 antibody levels were negatively associated with left ventricular ejection fraction (LVEF), both overall and within specific gCMP subtypes, including hypertrophic and arrhythmogenic cardiomyopathy. Additionally, anti-MYH6 antibody levels were negatively associated with right ventricular function, measured by tricuspid annular plane systolic excursion (TAPSE).

**Conclusion:**

Our findings stress the diagnostic potential of HRA profiling and indicate that gCMP patients might profit from therapeutic targeting of HRA.

**Supplementary information:**

The online version contains supplementary material available at https://doi.org/10.1186/s12872-026-06808-3.

## Introduction

Genetic cardiomyopathies (gCMP) represent a heterogeneous group of inherited heart muscle disorders caused by genetic variants in genes encoding structural and functional cardiac proteins. The principal subtypes include hypertrophic (HCM), dilated (DCM), arrhythmogenic right ventricular (ARVC), and restrictive cardiomyopathy (RCM).

Heart-reactive antibodies (HRA) have been detected in both healthy individuals and patients with cardiac conditions, yet their prevalence and clinical significance remain incompletely understood [1, 2]. Autoantibodies targeting cardiac Troponin I (cTnI) or Troponin T [TNNT2, Online Mendelian Inheritance in Man (OMIM): 191045] have been reported in 0–20% of the general population, in 7–22% of idiopathic DCM, and in up to 30% of ischemic cardiomyopathy patients [3, 4]. Similarly, autoantibodies against the beta-1 adrenergic receptor (β1AR) have been identified in approximately 19% of healthy individuals. Of note, those autoantibodies binding to the second extracellular loop (EC_II_) of the β1AR and activating the receptor, have been identified in around 40% of idiopathic DCM patients, where they are associated with adverse outcomes [5].

Tropomyosin 1 (TPM1, OMIM: 191010) is a fundamental component of the cardiac sarcomere, where it contributes to the regulation of actin–myosin interactions and, thereby, to the contractile function of cardiomyocytes. Genetic variants in *TPM1* that perturb the structural or functional properties of the encoded protein have been implicated in the development of cardiomyopathies, underscoring the importance of intact TPM1 for sarcomeric function and normal cardiac performance. IgG antibodies against Tropomyosin have been detected in up to 66% of idiopathic DCM patients, though lower prevalence rates (around 7%) have also been reported [6, 7]. In hypertrophic cardiomyopathies (HCM), the frequency of anti-TPM1 antibodies appears to be similar to that in the general population (approximately 15%) [8]. The Myosin Heavy Chain 6 (MYH6, OMIM: 160710) gene encodes the alpha-myosin heavy chain, primarily expressed in atrial tissue, and mutations in the gene are linked to atrial septal defects and sick sinus syndrome [9]. Myosin Heavy Chain 7 (MYH7, OMIM: 160760) encodes for the beta-myosin isoform, a key ventricular protein, and mutations in this gene are responsible for ~ 30% of HCM cases [10]. Anti-MYH6 antibodies, while of uncertain pathogenic significance, are found in 2% of healthy controls and up to 20% of idiopathic DCM patients [11]. While no extensive data exists regarding a specific pathophysiological role or prevalence of anti-Myosin Light Chain 7 (MYL7, OMIM: 613993) antibodies, the exclusive expression of MYL7 in cardiac tissue, as documented in the Human Protein Atlas, means that only cross-reactivity with other target proteins would lead to binding of anti-MYL7 antibodies to tissues other than the heart. This cardiac specificity was a key consideration for antigen selection, as antibody reactivity against MYL7 may provide a more tissue-specific readout of cardiac-directed autoimmunity and is less likely to reflect antibody binding related to extra-cardiac expression of the target antigen. In a large UK Biobank cohort of 200,643 individuals undergoing whole-exome sequencing, the prevalence of pathogenic or likely pathogenic variants associated with HCM, DCM, and ARVC was approximately 1:149, 1:251, and 1:578 [12].

The presence of HRA in gCMP suggests a potential autoimmune component in disease progression. However, previous studies lacking genetically defined cohorts, did not identify reactivities to defined (cardiac) antigens, and often omitted age- and sex-matched controls, which restricts their interpretability. To address these limitations, we employed bead-based flow cytometric assays to systematically analyze HRA against six defined cardiac antigens, including β1AR-EC_II_, TPI3, TPM1, MYH6, MYH7, and MYL7 in genetically confirmed gCMP patients and matched healthy controls. This antigen-specific, age-stratified approach enabled the differentiation between disease-associated and background antibody reactivity, thus allowing to explore associations between HRA patterns and clinical phenotypes. Based on HRA profiling, our study aimed to improve disease characterization, risk stratification, and to identify potential targets for the development of novel immunomodulatory strategies.

## Methods

### Patients and controls

This study was conducted as a retrospective case-control study to investigate the presence and clinical relevance of HRA in patients with gCMP. Eligibility required either a confirmed genetic mutation consistent with a gCMP phenotype, or the presence of such a variant in individuals with minor cardiac abnormalities. Variant classification followed the 2015 ACMG/AMP guidelines [13]. A subset of patients carried high-confidence variants of uncertain significance (VUS-high) [14]. Patients were consecutively clinically and genetically investigated through the “Center for Inherited Cardiovascular Diseases” at the University Hospital Würzburg. The study protocol was approved by the Ethics Committee of the Medical Faculty, Julius Maximilians University, Würzburg, Germany (vote #29/17). The study was conducted in accordance with the principles of the Declaration of Helsinki. All participants provided written informed consent prior to study participation.

Control participants were drawn from the prospective STAAB cohort (Characteristics and Course of Heart Failure Stages A-B and Determinants of Progression), a population-based, longitudinal cohort study of adults aged 30–79 years from Würzburg, Germany, free from heart failure at enrollment. The STAAB study protocol was approved by the Ethics Committee of the Medical Faculty, Julius Maximilians University, Würzburg, Germany (vote #98/13). The study was conducted in accordance with the principles of the Declaration of Helsinki [15]. All participants provided written informed consent prior to study participation. The detailed methodology has been published [16–18]. Controls were matched to patients (one control per patient) by age and sex from a subsample (*n* = 601 STAAB) in which HRA levels had previously been measured. For gCMP patients under 30 years of age, controls were selected from healthy CICD participants confirmed to be free of genetic variants or cardiomyopathy. After a pilot study comprising samples from 50 gCMP patients and 50 healthy controls suggested that both groups might, indeed, differ regarding their HRA profiles, we decided to double the number of samples analyzed to generate a better data basis for statistical analyses.

### Antibody detection and quantification

We used bead-based assays for flow cytometric detection of autoantibodies against defined (cardiac) antigens established by us [19]. For these, magnetic beads were covalently coated with sarcomeric proteins or a peptide resembling the EC_II_ of the β1AR. To detect antibody binding, ninety-six well plates were used and the beads were first blocked with 10% bovine serum albumin (BSA) in phosphate-buffered saline (PBS) to prevent non-specific binding. Antibody reactivities to the coated beads were subsequently quantified from serum samples preserved at -80 °C. To avoid bias, the samples were measured by researchers blinded to the sex and age of the patients/ study participants the samples had been obtained from. After incubation with patient serum, primary antibodies specific to each sarcomeric protein or the β1AR-EC_II_ peptide were captured on the bead surface. Bound antibodies were detected with Alexa Fluor 647-conjugated AffiniPure goat anti-human IgG (H + L) (Jackson ImmunoResearch). To ensure that the coated beads used for antibody detection were of sufficient quality, each assay included a positive control containing a primary antibody recognizing the coated protein. Additionally, a second control was used comprising human immunoglobulin G (IgG_4_, Kappa, from human myeloma plasma, Sigma-Aldrich) coated-beads capable of directly binding the secondary antibody. Samples were measured on a FACSCalibur flow cytometer (BD Biosciences), and data analyzed with FlowJo software (FlowJo, LLC, Ashland, OR, USA). Antibody binding was quantified as the ratio of median fluorescence intensity (MFI) between antigen-coated and uncoated control beads. The assay was designed to capture subtle variations in antibody binding, and robustness of the data was ensured by running each sample in duplicates. The investigated antigens encompassed the β1AR-EC_II_ peptide (custom-made, Peptide specialty laboratories GmbH) or a ‘scrambled’ control (custom-made, Peptide specialty laboratories GmbH), TPI3 (Recombinant Human Cardiac Troponin I, Abcam), MYL7 (Recombinant Human MYL7, Abcam), TPM1 (Recombinant Human Tropomyosin-1, Prospec), MYH6 (Recombinant Myosin Heavy Chain 6, Cardiac Muscle, Alpha (MYH6), Cloud-Clone Corp.), and MYH7 (Myosin Heavy Chain 7, Cardiac Muscle, Beta (MYH7), Cloud-clone Corp.) [17]. Antigen selection was guided by their documented clinical relevance in cardiomyopathies and expression in human cardiac tissue, as documented in the Human Protein Atlas and further outlined below. Anti-β1AR-EC_II_ antibodies were included due to their potential to stimulate the β1-adrenergic receptor leading to sustained signaling in DCM, while anti-TPI3 antibodies have also been detected in both DCM and HCM previously and may indicate disease-associated cardiomyocyte injury. In hypertrophic cardiomyopathy, mutations affecting TPM1 and TPI3, as well as MYH6 and MYH7, are well recognized. Notably, MYL7 is exclusively expressed in cardiac tissue, meaning that antibodies recognizing MYL7 should most faithfully reflect anti-cardiac autoimmunity.

### Data analysis

Continuous variables were expressed as mean ± standard deviation (SD) or median with interquartile range (IQR) based on data distribution. Categorical variables were presented as absolute numbers and percentages. Group comparisons were performed using Student’s t-test, Mann-Whitney U test or Kruskal-Wallis test for continuous variables, and chi-square or Fisher’s exact test for categorical variables. Associations between HRAs and clinical parameters were assessed using linear regression or correlation analysis by Spearman´s rank -correlation, as appropriate. A p-value < 0.05 was considered statistically significant. SPSS Statistics, Version 29.0 (IBM) was used for statistical analyses.

## Results

### Study population

To study the prevalence and possible clinical impact of HRA in gCMP, we retrospectively analyzed samples from a total of 100 patients (mean age 46.4 ± 15.9 years, 53% women) with confirmed genetic variants linked to gCMP and 100 age- and sex-matched healthy controls. The gCMP patient samples comprised individuals with HCM (*n* = 44), DCM (*n* = 32), ARVC (*n* = 21), RCM (*n* = 3) (Table 1). Most patients presented in an outpatient setting with mild compensated heart failure symptoms (NYHA functional class I or II). Among gCMP patients, 58% had a family history of cardiomyopathy or arrhythmias, while 37% reported a family history of sudden cardiac death. The majority of patients (84%) harbored pathogenic or likely pathogenic variants, while 16% carried a high-confidence variant of uncertain significance (VUS-high).

**Table 1:** Data are presented as mean (standard deviation) for continuous variables and n (%) for categorical variables. Percentages are rounded to whole numbers. Data completeness was available in ≥90% for all variables, with the following exceptions: LVEF (87%) and coronary heart disease (87%) among healthy participants; NYHA I–III (88%) and HFpEF/HFmrEF/HFrEF (88%) among gCMP patients

	gCMP *n* = 100	Healthy *n* = 100
**Age** (years)	46.4 (15.9)	48.2 (15.9)
**Sex** (Female/Male)	53 (53%) / 47 (47%)	53 (53%) / 47 (47%)
**Family history of gCMP or arrhythmias**	58 (58%)	n/a
**Family history of SCD**	37 (37%)	n/a
**Type of genetic variant: Pathogen/Likely pathogen/VUS**	48 (48%) / 35 (35%) / 17 (17%)	n/a
**Sarcomer/Desmosomal/Other**	56 (56%) / 21 (21%) / 22 (22%)	n/a
**NYHA I-III**	32 (37%) / 33 (38%) / 23 (25%)	n/a
**Coronary artery disease**	11 (11%)	2 (2%)
**Type of Cardiomyopathy** (ARVC/DCM/HCM/RCM)	21 (21%) / 32 (32%) / 44 (44%) / 3 (3%)	n/a
**Time from first diagnosis (**years**)**	4.3	n/a
**Implantable cardioverter defibrillator**	42 (42%)	n/a
**NT-proBNP** (pg/ml)	983 (2.284)	n/a
**LVEF** (%)	57 (11.5)	61 (3.8)
**HFpEF/HFmrEF/HFrEF**	65 (74%) / 11 (13%) / 11 (13%)	n/a

Abbreviations: ARVC, arrhythmogenic right ventricular cardiomyopathy; DCM, dilated cardiomyopathy; gCMP, genetic cardiomyopathy; HCM, hypertrophic cardiomyopathy; HFmrEF, heart failure with mildly reduced ejection fraction; HFpEF, heart failure with preserved ejection fraction; HFrEF, heart failure with reduced ejection fraction; LVEF, left ventricular ejection fraction; n/a, not available; NYHA, New York Heart Association classification of heart failure severity; RCM, restrictive cardiomyopathy; SCD, sudden cardiac death; VUS, variant of uncertain significance

The mean time since diagnosis was 4.3 years, with heart failure with preserved ejection fraction (HFpEF) being the predominant HF subtype, affecting 74% of patients (Table 1). The mean left ventricular ejection fraction (LVEF) was 57 ± 11.5%, with 13% classified as heart failure with mildly reduced ejection fraction (HFmrEF), and 13% as heart failure with reduced ejection fraction (HFrEF). The mean NT-proBNP level was 983 pg/ml ± 2.284 pg/ml, and 42% of patients had an implantable cardioverter defibrillator (ICD).

The mean age of control participants was 48.2 ± 15.9 years (53% women), and none of them had a diagnosis of heart failure, a confirmed cardiomyopathy, or a confirmed predisposing genetic mutation.

### The unstratified cohorts of gCMP patients and controls differed in anti-TPI3 and anti-TPM1 antibody levels

**Fig. 1:**
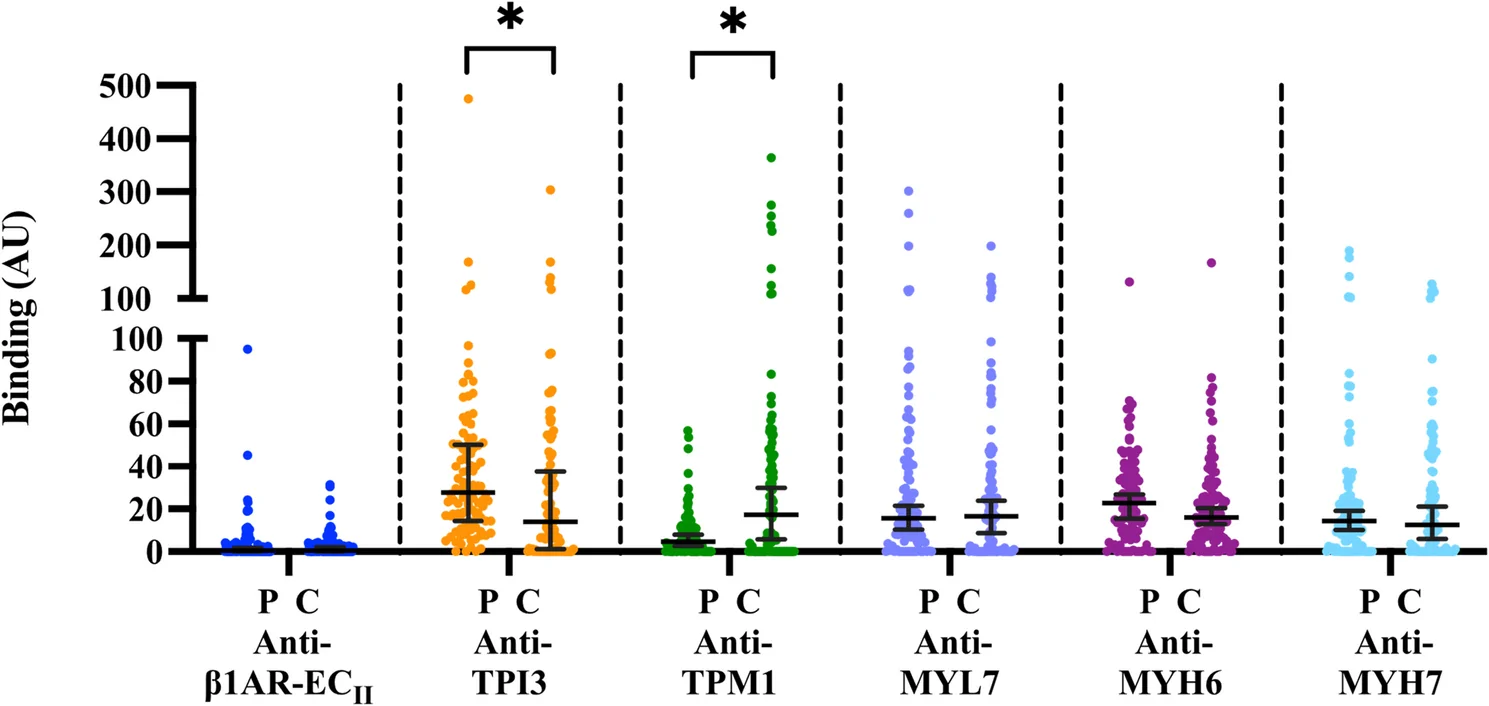
Antibody binding levels in patients with genetic cardiomyopathies (gCMP) compared to healthy controls. Anti-TPI3 binding levels were higher (*p*≤0.001), whereas anti-TPM1 binding levels were lower in gCMP patients compared to controls (*p*≤0.001). Further, a trend towards higher anti-Myosin Heavy Chain 6 (MYH6) binding was observed in gCMP patients (*p*=0.085). Mann-Whitney U test: *p<0.05. Number of observations: n=100 for each column. P=Patients, C=Controls, β1AR-ECII = Second extracellular loop of the β1 adrenergic receptor, TPI3= Troponin 3, TPM1 = Tropomyosin 1, MYL7 = Myosin light chain 7, MYH6 = Myosin heavy chain 6, MYH7 = Myosin heavy chain 7

We used novel bead-based assays to assess HRA profiles in samples from gCMP patients versus matched healthy controls [17]. The HRA profile comprised six cardiac antigens which we had picked according to their expression patterns as outlined in the Human Protein Atlas. Overall antibody reactivities against cardiac antigens varied considerably across individuals. Compared to healthy controls, gCMP patients exhibited significantly elevated antibody levels against TPI3 (Fig. 1, orange dots) when analyzed across all age groups. In contrast, anti-TPM1 antibody levels were significantly lower in gCMP patients compared to healthy controls in the overall cohort (Fig. 1, green dots), while this effect mainly seemed to be driven by the 31–40-year age subgroup. No significant differences were observed for antibodies against MYH6, MYH7, MYL7, or β1AR-EC_II_ (Fig. 1). Therefore, while HRA were readily detectable both in gCMP patients and healthy controls, HRA levels of anti-TPI3 and anti-TPM1 antibodies differed between both cohorts. Overall, autoantibody levels showed distinct age-dependent patterns, with anti-TPI3 remaining consistently elevated in gCMP patients compared to controls across most age groups, while anti-TPM1 differences tended to be higher in the control cohort and was mainly influenced by the 31-40-year age group.

### Elevation of anti-TPI3 antibody levels in gCMP patients compared to controls is age-dependent

For some autoantibodies, like anti-nuclear autoantibodies [20], the prevalence and titers increase with age, even in healthy individuals. Therefore, we also studied the impact of age on HRA in gCMP patients and healthy controls.

**Fig. 2:**
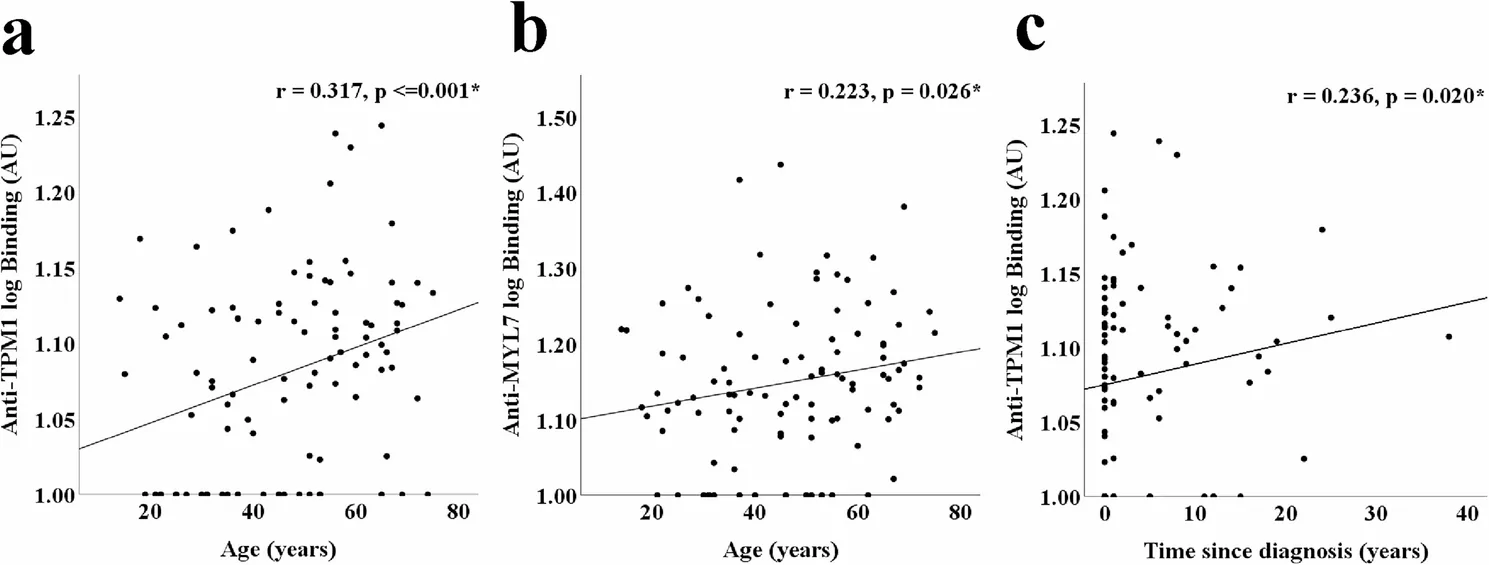
Age-dependent dynamics of heart-reactive antibodies (HRA) in gCMP patients. In gCMP patients, (**a**) anti-TPM1 binding demonstrated a significant increase with advancing age (*p*=0.001), while (**b**) anti-MYL7 binding also showed a pronounced age-related rise (*p*=0.026). **c **Anti-TPM1 antibody levels also showed an elevation over the observation period (*p*=0.020). To achieve a more uniform data distribution, binding values were transformed using the formula: log₁₀(√Binding + 102). Spearman´s rank -correlation: *p<0.05. Number of observations: n=100 for each graph

**Fig. 3:**
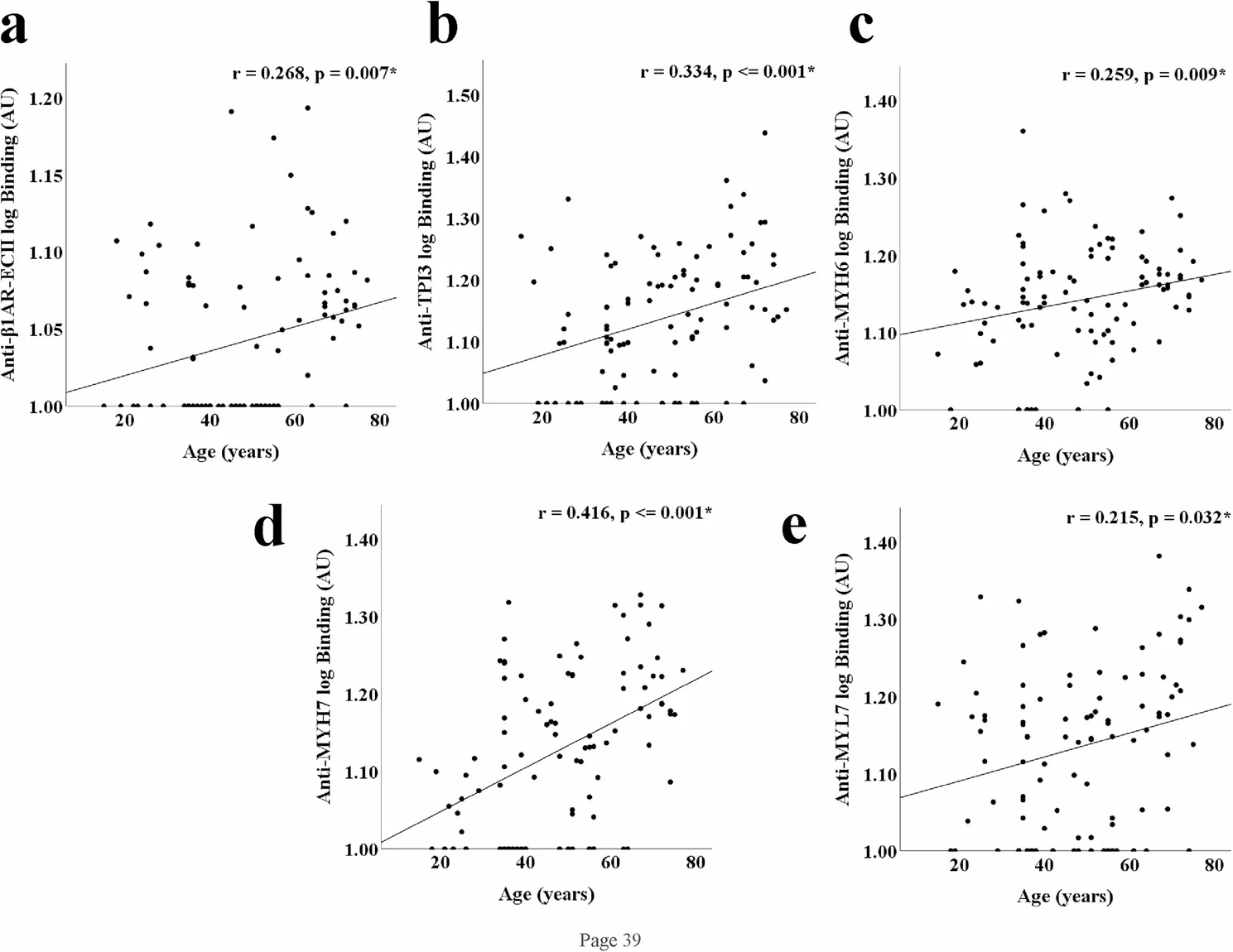
Age-dependent dynamics of HRA in healthy controls. In the control cohort, (**a**) Anti-β1AR-ECII peptide antibody binding showed an age-related enhancement (*p*=0.007). **b** Anti-TPI3 antibody binding displayed a consistent positive correlation with age (*p*≤0.001). **c** Moreover, MYH6 revealed an age-dependent increase (*p*=0.009). **d** Similarly, anti-MYH7 antibody binding exhibited a positive association with increasing age (p=0.001). **e** Lastly, anti-MYL7 also increased with advancing age (*p*=0.032). To achieve a more uniform data distribution, binding values were transformed using the formula: log₁₀(√Binding + 102). Spearman´s rank -correlation: *p<0.05. Number of observations: n=100 for each graph

**Fig. 4:**
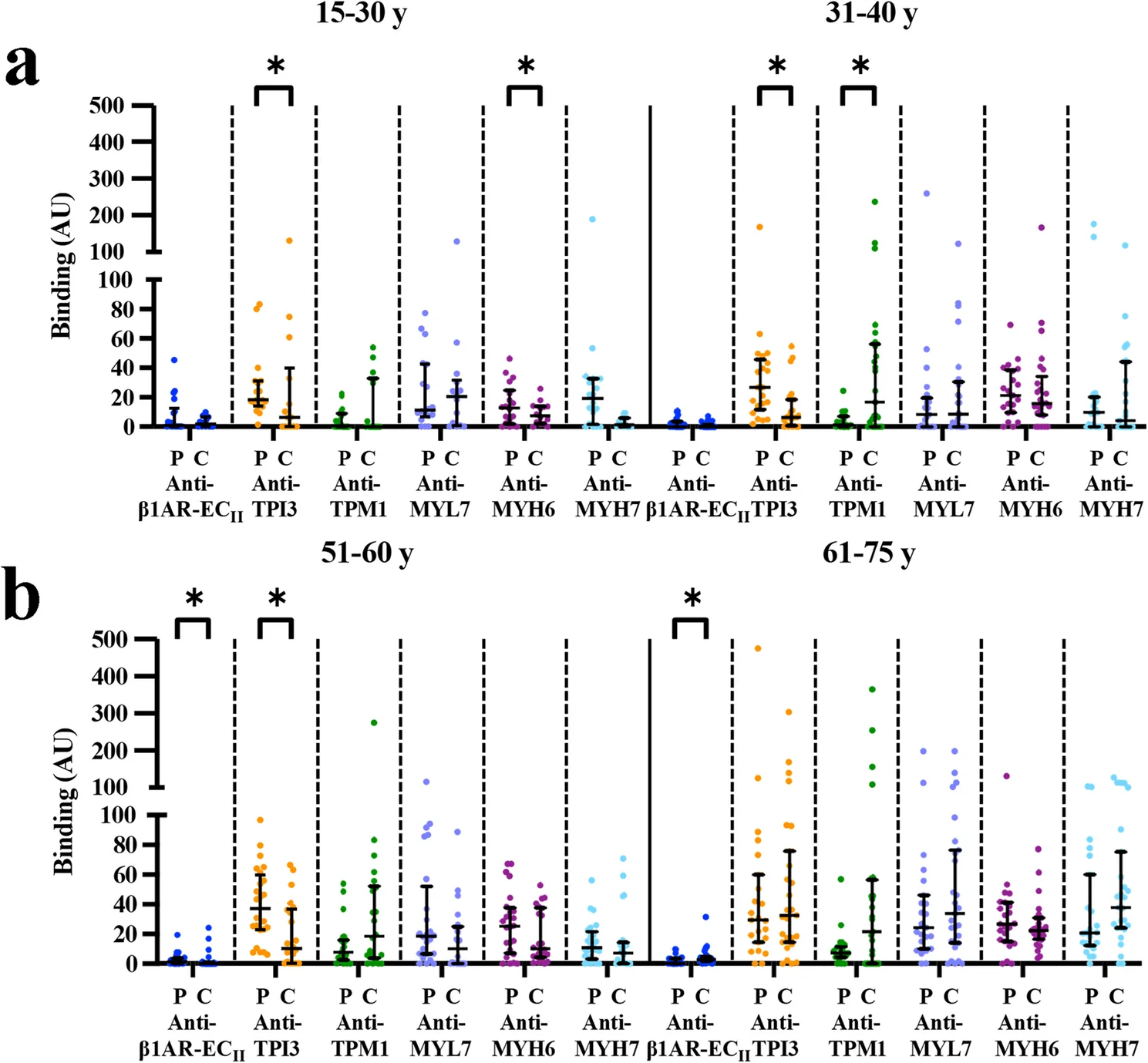
Age-specific antibody binding differences in gCMP patients and controls. **a** Among individuals aged 15–30 years, anti-TPI3 and anti-MYH6 antibody binding levels were elevated in gCMP patients compared to controls. Among individuals aged 31–40 years, anti-TPI3 antibody binding was elevated, while anti-TPM1 binding was markedly reduced in the gCMP cohort. **b** Among individuals aged 51–60 years, anti-β1AR-ECII antibody and anti-TPI3 antibody binding levels were higher in gCMP patients. In the highest age group, i.e. individuals aged 61–75years, anti-β1AR-ECII antibody binding was lower in gCMP patients compared to controls. Mann-Whitney U test: *p<0.05. Number of observations: 15-30y: *n*=18 in P, *n*=14 in C, 31-40y: *n*=21 in P, *n*=25 in C, 51-60y: *n*=24 in P, *n*=22 in C, 61-75y: *n*=23 in P, *n*=27 in C

In the gCMP cohort, age was positively associated with anti-TPM1 and anti-MYL7 antibodies (Fig. 2a-b).Notably, anti-TPM1 antibody levels not only increased with age, but also rose significantly following initial diagnosis (Fig. 2c). In healthy controls, antibody levels against β1AR-EC_II_, TPI3, MYH6, MYH7 and MYL7 showed positive association with age (Fig. 3a-e) with the most pronounced effect for anti-MYH7 autoantibodies (Fig. 3d). Comparing gCMP patients and healthy controls as a whole, we had observed higher anti-TPI3 antibody levels in patients (Fig. 1, orange dots). Stratification by age revealed that the overall differences in anti-TPI3 antibody levels were consistently present across all age groups except for samples from patients aged 61–75 years where there was no difference between gCMP patients and healthy controls (Fig. 4, orange dots). Regarding anti-TPM1 antibodies, the comparison of the total cohorts of gCMP patients and healthy controls had shown lower anti-TPM1 autoantibodies in gCMP patients compared to healthy controls (Fig. 1, green dots). Here, differences were only statistically significant for samples from individuals in the 31–40-year age group (Fig. 4a, green dots) with similar trends, however, for patients older than 40 years (Fig. 4b, green dots). For individuals aged 15–30 years, detection of anti-TPM1 antibodies was rare both in gCMP patients as well as healthy controls (Fig. 4a, green dots).

### Sex specific differences between gCMP patients and controls in anti-TPI3, TPM1 and MYH6 antibody levels

**Fig. 5:**
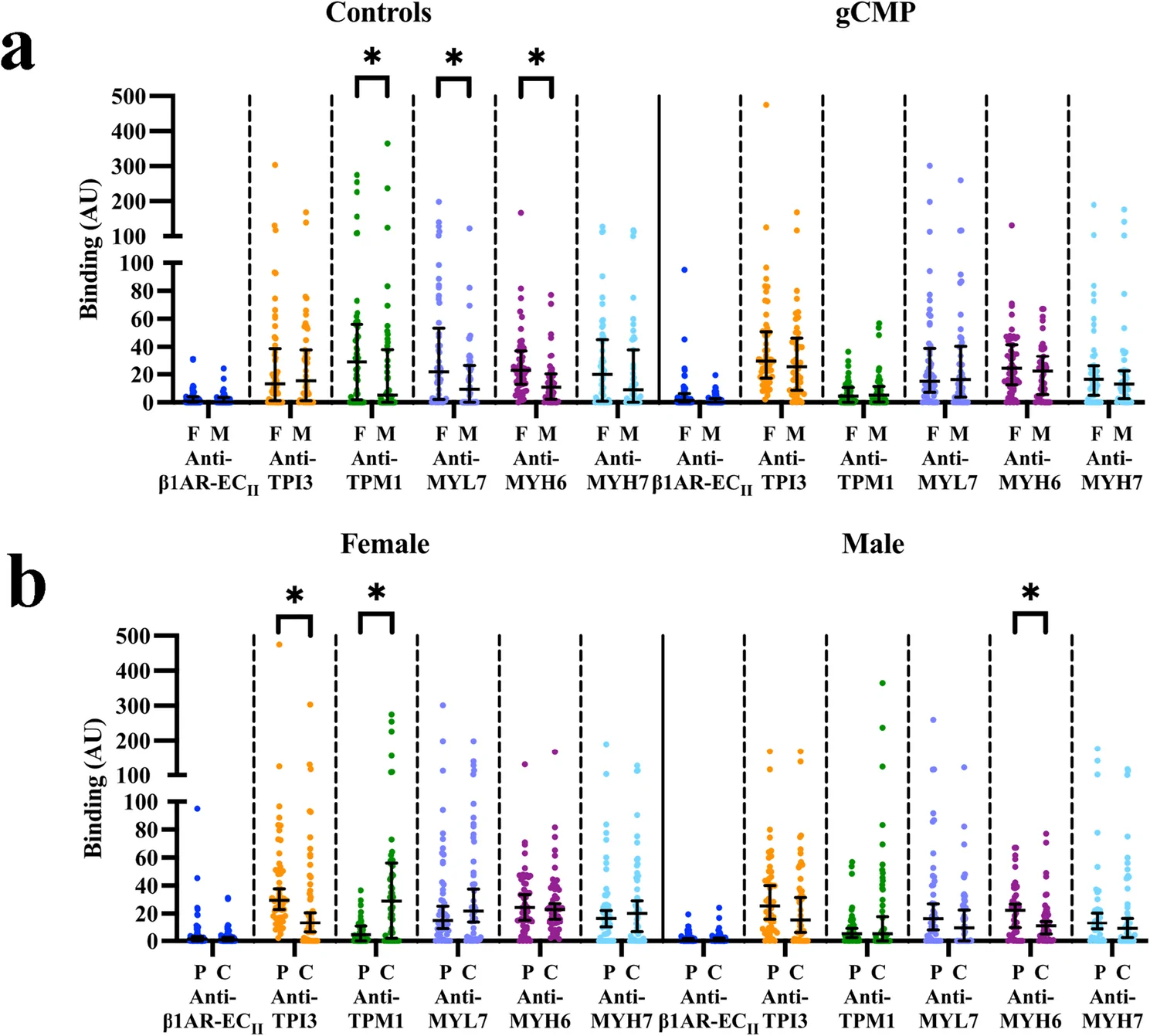
Sex-specific variations in antibody binding between gCMP patients and controls. **a** Antibody binding levels in healthy controls, stratified by sex. Female controls demonstrated higher binding levels for anti-TPM1, anti-MYL7 and anti-MYH6 antibodies compared to male controls. Antibody binding levels in the gCMP cohort stratified by sex revealed no sex-specific differences. **b** Comparison of antibody binding levels between female gCMP patients and controls. Female patients exhibited significantly higher anti-TPI3 but notably lower anti-TPM1 antibody levels compared to healthy controls. Analysis of antibody binding in male individuals revealed elevated levels for anti-MYH6 antibody levels in the gCMP patient cohort compared to controls. Mann-Whitney U test: *p<0.05. Number of observations: (**a**) *n*=53 for each column in F, *n*=47 for each column in M, (**b**) *n*=53 for each column in Female, *n*=47 for each column in Male

Stratification of samples from healthy controls according to sex showed that higher anti-TPM1, MYL7 and MYH6 antibody reactivities were observed in female compared to male participants (Fig. 5a, left). In contrast, there were no differences in HRA levels between male and female gCMP patients (Fig. 5a, right). When further only considering female participants, there was the same pattern of elevated anti-TPI3 and reduced anti-TPM1 antibody levels in gCMP patients compared to healthy controls as seen for the overall cohort (Fig. 5b, left). This was not the case for male participants where the differences regarding anti-TPI3 and anti-TPM1 antibodies were far less pronounced (Fig. 5b, right). But the isolated analysis of samples from male donors showed that gCMP patients had higher anti-MYH6 autoantibody concentrations compared to healthy controls (Fig. 5b, right, pink dots), which was not the case for the overall cohort (Fig. 1, pink dots).

Together, both age and sex significantly influenced HRA expression in both groups.

### Anti-MYH6 and TPM1 antibody levels negatively associated with cardiac function in gCMP patients

**Fig. 6:**
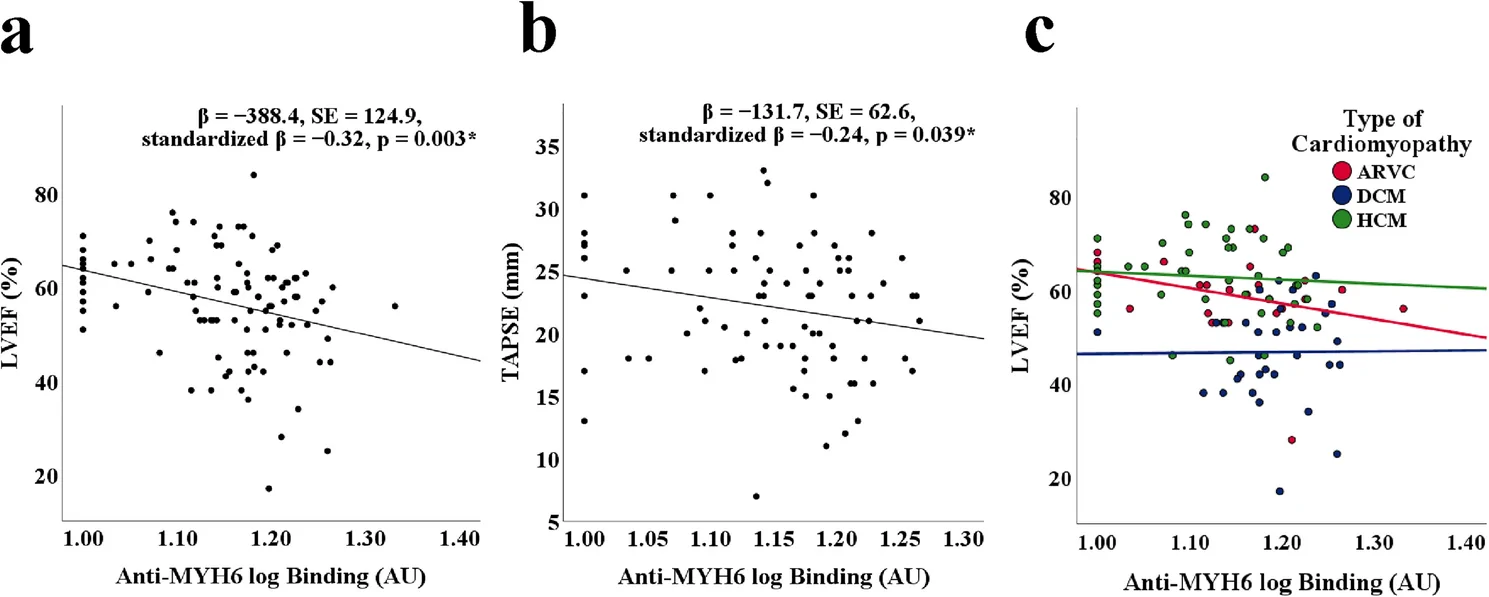
Inverse association between anti-MYH6 binding and left ventricular function. **a **Scatter plot illustrating the inverse relationship between left ventricular ejection fraction (LVEF) and anti-MYH6 antibody binding levels (p=0.003). **b** Right ventricular function, measured echocardiographically by TAPSE, decreased with increasing anti-MYH6 antibody binding levels (p=0.039). **c** The scatter plot from panel **a** was further stratified by cardiomyopathy subtype. Significant interactions between MYH6 antibody level and cardiomyopathy subtype were observed (ARVC vs DCM: *p* ≤ 0.001; HCM vs DCM: *p* ≤ 0.001; DCM as reference). After correction for age and sex differences remained significant. To achieve a more uniform data distribution, anti-MYH6 antibody binding values were transformed using the formula: log₁₀(√Binding + 102). **a**, **b** Linear regression models: *p<0.05, (**c**) multivariate linear regression model. Number of observations: **a**, **c** *n*=87 for each graph, (**b**) *n*=75

**Fig. 7:**
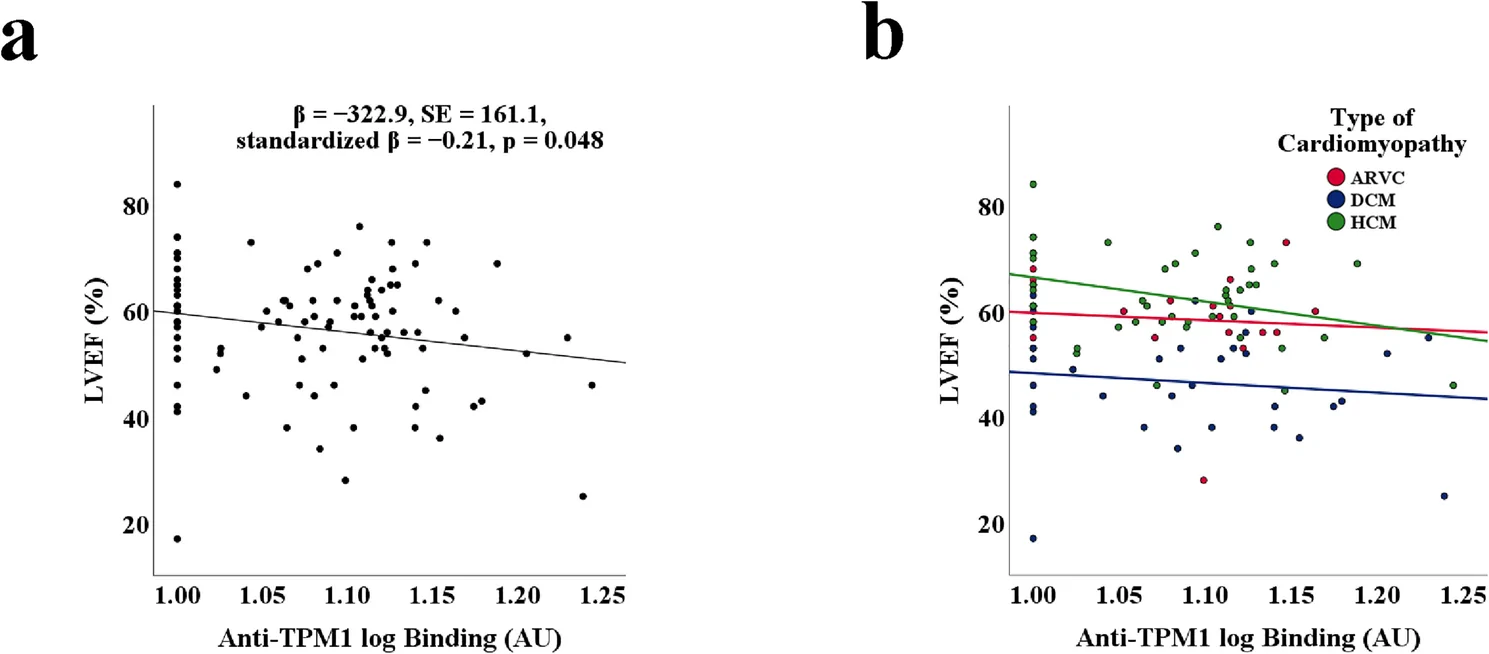
Inverse association between anti-TPM1 binding and left ventricular function. **a** Scatter plot depicting the negative association between LVEF and anti-TPM1 antibody binding levels. Data were normalized using the transformation: log₁₀(√TPM1 Binding + 102), to ensure a more balanced distribution (p=0.048). **b** Significant interactions between TPM1 antibody level and cardiomyopathy subtype were observed (ARVC vs DCM: *p* ≤ 0.024; HCM vs DCM: *p* ≤ 0.009; DCM as reference). After correction for age and sex differences remained significant. **a** Linear regression model: *p<0.05, (**b**) multivariate linear regression model. *p<0.05. Number of observations:* n*=87 for each graph

To obtain insights into whether some of the HRA measured by us might be associated with the clinical severity of gCMP, we associated HRA levels with clinical indices of cardiac function. Anti-MYH6 antibody levels negatively associate both with left ventricular ejection fraction (LVEF) (*r*=-0.320, *p* = 0.003) and, reflecting right heart function, tricuspid annular plane systolic excursion (TAPSE) (*r*=-0.239, *p* = 0.039) (Fig. 6a, b). Similarly, anti-TPM1 antibodies showed an inverse association with LVEF (*r*=–0.213, *p* = 0.048) (Fig. 7a). No significant associations were observed for anti-TPI3, MYH7, MYL7, and β1AR-EC_II_ antibody levels and cardiac function (data not shown). Conversely, in HCM anti-TPM1 antibody levels were inversely associated with LVEF (Fig. 7b).

Analysis by cardiomyopathy subtype revealed significant antibody-by-subtype interactions, indicating that the relationship between anti-MYH6/anti-TPM1 levels and LVEF varies across the respective cardiomyopathy especially when comparing ARVC and HCM toward DCM. (Figures 6c and 7c).

In contrast to these links of antibody levels and cardiac function, neither NT-proBNP concentrations in blood nor NYHA functional class, except for modestly higher anti-TPM1 antibody levels in gCMP patients with NYHA class II compared to patients with class I or III (Suppl. Fig. S1), seemed to be associated with antibody levels. Additionally, increased anti-MYH6 antibody levels were observed in gCMP patients with HFrEF compared to those with HFpEF (Suppl. Fig. S2). The only antibodies that showed a significant increase when stratifying samples for the cellular substructure of the primary antigen associated with the respective genetic variant were anti-MYH6 antibodies, which were more elevated in patients with non-sarcomeric, non-desmosomal (“other”) gene variants compared to those with sarcomeric or desmosomal gene variants (Suppl. Fig. S3).

Thus, among gCMP patients, only anti-MYH6 and anti-TPM1 antibody levels were associated with clinical indices of cardiac function.

### Antibody binding across genetic subgroups in the gCMP cohort

Antibody binding levels were compared according to the underlying genetic variant across the different cardiomyopathy subtypes. Among the 98 patients with an identified genetic variant, MYH7 was the most frequently observed variant (*n* = 19), followed by MYBPC3 (*n* = 17), PKP2 (*n* = 12), FLNC (*n* = 11), and TTN (*n* = 10) (Supplementary Fig. S4). The distribution of genetic variants differed substantially across the cardiomyopathy phenotypes. MYH7 and MYBPC3 variants were predominantly observed in patients with HCM, accounting for 13 and 17 cases, respectively. In contrast, PKP2 variants were exclusively identified among patients with ARVC (*n* = 12), whereas TTN variants were predominantly associated with DCM (*n* = 8). DSP variants were distributed across ARVC, DCM, and RCM, while FLNC variants were observed across all four cardiomyopathy subtypes. Less frequently observed variants included LMNA, TNNC1, TNNT2, TNNI3, TPM1, ACTC1, DSG2, and MYL3. When antibody binding was compared between patients carrying different genetic variants, a statistically significant difference for antibody levels was observed only for MYH6 (*p* = 0.047). For the other analyzed proteins, no significant differences in antibody binding according to the underlying genetic variant were detected (Supplementary Fig. S5). Proteins and antibodies used in the bead based assay were summarized (Supplementary Fig. S6/S7).

## Discussion

In this study, we profiled HRA in a well-characterized cohort of patients with gCMP, comparing them with age- and sex-matched healthy controls. Using bead-based flow cytometric assays comprising six functionally relevant cardiac antigens [18], we observed both elevated and diminished antibody reactivities in gCMP patients. Furthermore, levels of autoantibodies against MYH6 or TPM1 were inversely associated with ventricular function, reflected by reduced LVEF and TAPSE.

Previous studies investigating cardiac autoantibodies in cardiomyopathy have largely focused on individual antigens or on specific phenotypes, often without genetic confirmation or age- and sex-matched controls. Our study went beyond the available literature by employing a high-resolution antigen-specific approach in a genetically and clinically stratified patient population.

gCMP patients showed elevated anti-TPI3, but reduced anti-TPM1 antibody reactivity compared to controls, with age positively associated with antibody levels against almost all antigens in controls and certain antigens in gCMP patients (Figs. 1, 2 and 3). The elevated anti-TPI3 antibody reactivity in gCMP patients may reflect enhanced stimulation of the immune system by cardiac antigens due to structural damage or mutations. However, there was no preferential induction of HRA in gCMP patients with sarcomeric mutations (Suppl. Fig. S3), implying that there is no direct link between HRA expression and the underlying mutation(s). Still, future work should assess antibody responses against mutated cardiac proteins. Troponin-targeting antibodies are responsible for DCM in PD-1 deficient mice [21], and for cardiomyopathy patients in general, anti-troponin antibodies have been reported in up to 22%, compared to 20% of healthy individuals [3]. With our bead-based FACS assays the proportion of samples in which anti-TPI3 antibodies were detected was 78% in healthy controls versus 97%, respectively, when considering any binding above zero as a positive signal. A limitation here, of course, is the lack of standardization and cut-off definitions, reducing comparability across studies. Moreover, different studies used different assay types to detect HRA: ELISA is sensitive, but results vary for kits from different manufacturers and sometimes even batch; Western blot confirms specificity but is laborious and poor in detecting antibodies recognizing conformational epitopes; chemiluminescence assays offer high sensitivity but require costly devices; lateral flow tests are fast but lack quantification. The bead-based FACS assays as used by us have a wide dynamic range and allow for multiplexing but require expensive kits and technical expertise.

Previously, anti-tropomyosin autoantibodies have been reported in up to 66% of DCM patients suggesting that they were more prevalent in DCM than in non-DCM patients, and healthy controls [7]. This is in contrast to the reduced anti-TPM1 antibody levels we detected in gCMP patients compared to healthy controls. Differences in the assay systems used and, as already mentioned, definition of cut-offs, could account for the seemingly discrepant results. As we relied on ratios of reactivities to antigen- versus uncoated beads, determined for each patient serum and antigen individually, we avoided having to set (arbitrary) cut-off values. Lower anti-TPM1 antibody levels in gCMP patients compared to healthy controls may result from a lack of induction of these antibodies in gCMP patients, deletion or exhaustion of TPM1-specific B cells [22], or induction of regulatory B cells [23] in gCMP patients due to repeated exposure to TPM1 as an autoantigen [24, 25]. It remains unclear whether anti-TPM1 antibodies in healthy individuals are natural, shaped by commensals, or are induced by infections/vaccinations. Functionally, these antibodies might limit myocardial damage and reduce risk - similar to what has been suggested for coronary heart disease [26–28] - and the reduced prevalence of potentially protective anti-TPM1 antibodies in gCMP patients might promote disease progression.

At first glance, sex differences were only present in healthy controls (Fig. 5a), to which estrogen and testosterone may contribute [29, 30]. At second glance, stratifying the data obtained with sera from gCMP patients revealed sex-specific differences compared to healthy controls (Fig. 5b). Our findings underscore the influence of sex on HRA expression, a variable often overlooked in earlier studies. This highlights that considering sex when further studying the impact of HRA on the progression of gCMP is crucial, and might constitute a first step to “personalized” medicine in cardio-immunology.

Our analyses revealed age-related changes in HRA levels (Figs. 2, 3 and 4), which is in line with findings linking repeated antigen exposure during aging with autoantibody elevation [31–33]. However, for anti-TPI3 antibodies, clearly elevated in gCMP patients compared to healthy controls, there was no increase with age in patients, but in healthy controls. This has implications for risk stratification in older patients.

The inverse association between anti-MYH6 and anti-TPM1 antibody levels and ventricular function are among the most clinically salient findings of this study [34, 35]. MYH6, primarily expressed in atrial tissue but also transiently in the ventricles during development, may become antigenically exposed in disease states, eliciting an autoimmune response. Prior work has suggested that MYH6-reactive antibodies can impair cardiac conduction and contribute to atrial pathology. Our data now extend this potential relevance to left ventricular and right heart dysfunction, particularly in ARVC. Altered MYH6 expression, mutations or MYH6/MYH7 imbalances disrupt sarcomeric stability and contractile efficiency, which may, thus, directly lead to ventricular dysfunction [9]. Moreover, a decrease in TAPSE and a shift in the MYH6/MYH7 ratio have been reported to be associated with downregulated expression of MYH6 and enzymes required for fatty acid oxidation (FAO) in response to volume overload. Reduced FAO enzyme activity suggests impaired energy metabolism in the myocardium, potentially leading to inadequate ATP production needed for efficient contraction, especially within the right ventricle [36]. After cardiomyocyte damage or death, MYH6 expressed intracellularly might become accessible to anti-MYH6 antibodies that may then promote cytotoxicity and/or immune activation [37, 38]. TPM1 plays a critical role in regulating actin-myosin interactions by modulating calcium sensitivity during sarcomeric contraction [39]. Mutations or disruptions in TPM1, as seen in HCM, lead to hypercontractility [40]. As for anti-MYH6 antibodies, it is, however, very unlikely that anti-TPM1 antibodies are capable of directly interfering with cardiomyocyte viability or function. Rather, anti-TPM1 antibodies will be able to bind to their intracellular target protein once there already is substantial damage or death of cardiomyocytes. However, the inverse associations observed between anti-MYH6/anti-TPM1 antibody levels and ventricular function do not establish a causal relationship. Given the predominantly intracellular localization of these sarcomeric proteins, their limited accessibility to circulating immunoglobulins in intact cardiomyocytes suggests that these antibody responses may primarily reflect secondary antigen exposure and release following myocardial injury rather than a direct pathogenic autoimmune mechanism.

While our method ensures robustness through controls and signal normalization, and provides compelling evidence linking HRAs to gCMP, several limitations of our study must be acknowledged. First of all, the cross-sectional nature of this study limits causal inference, and our findings require validation in larger, longitudinal cohorts. Importantly, while some HRAs may be pathogenic, others might represent epiphenomena or even protective responses. Second, although our sample size allowed for exploratory analyses, the limited number of patients within individual cardiomyopathy subgroups, particularly RCM, restricts statistical power for subgroup-specific analyses and limits the generalizability of our findings. The same applies to the analysis of individual genetic variants, for which the number of patients within specific genetic subtypes was also very limited. Another limitation in the interpretation of our findings is the lack of systematic genetic screening for gCMP-associated pathogenic or likely pathogenic variants in the control cohort, except in individuals with a clear clinical indication for genetic testing. Therefore, the presence of undetected genetic variants associated with cardiomyopathy cannot be completely excluded and may represent a potential source of residual confounding. Although normalization to uncoated beads provides a standardized measure of antibody binding within our experimental setup, the absence of established reference thresholds limits direct cross-study comparability and the potential clinical translation of our findings. Further assay standardization and validation in independent cohorts will therefore be required. Moreover, the clinical relevance of HRAs may vary by antigen, disease stage, and genetic background—factors that future research should address. Despite these limitations, our study provides important implications for clinical practice and future research. Clinically, our findings suggest that profiling HRA may provide additional insights into disease state and progression in gCMP.

## Conclusion

In summary, this study underscores significant associations between HRA levels and key clinical characteristics in patients with gCMP, including age, sex, and echocardiographic indices of cardiac function. Notably, age and sex emerged as important modulators of HRA expression. Our observations are hypothesis-generating and warrant validation in larger, longitudinal cohorts. Future research should also explore the mechanistic roles of HRAs in disease progression and their potential utility as biomarkers or therapeutic targets in personalized management strategies for gCMP. 

## Supplementary information

Below is the link to the electronic supplementary material.

Supplementary Material 1

## Data Availability

The data underlying this article will be shared upon reasonable request to the corresponding author, in accordance with applicable ethical and data protection regulations. No additional data are publicly available.

## Abbreviations

ANA: Anti-nuclear antibody
ARVC: Arrhythmogenic right ventricular cardiomyopathy
ATP: Adenosine triphosphate
β1AR-ECII: Second extracellular loop of the β1-adrenergic receptor
BSA: Bovine serum albumin
CHFC: Comprehensive Heart Failure Center
CICD: Center for Inherited Cardiovascular Diseases
CLIA: Chemiluminescence immunoassay
DCM: Dilated cardiomyopathy
ELISA: Enzyme-linked immunosorbent assay
FAO: Fatty acid oxidation
FACS: Fluorescence-activated cell sorting
FcRn: Neonatal Fc receptor
gCMP: Genetic cardiomyopathy
HCM: Hypertrophic cardiomyopathy
HF: Heart failure
HFmrEF: Heart failure with mildly reduced ejection fraction
HFpEF: Heart failure with preserved ejection fraction
HFrEF: Heart failure with reduced ejection fraction
HRA: Heart-reactive antibody
ICD: Implantable cardioverter defibrillator
IgG: Immunoglobulin G
IQR: Interquartile range
LVEF: Left ventricular ejection fraction
MFI: Median fluorescence intensity
MYH6: Myosin heavy chain 6
MYH7: Myosin heavy chain 7
MYL7: Myosin light chain 7
NT-proBNP: N-terminal pro B-type natriuretic peptide
NYHA: New York Heart Association
OMIM: Online Mendelian Inheritance in Man
PBS: Phosphate-buffered saline
RCM: Restrictive cardiomyopathy
SD: Standard deviation
SCD: Sudden cardiac death
STAAB: Characteristics and Course of Heart Failure Stages A-B and Determinants of Progression (cohort study)
TAPSE: Tricuspid annular plane systolic excursion
TNNT2: Troponin I type 2 (cardiac)
TPI3: Troponin I3
TPM1: Tropomyosin 1
VUS: Variant of uncertain significance
VUS-high: High-confidence variant of uncertain significance
