# Supplementary material for: Heart-reactive antibody levels differentially associate with ventricular function in hereditary cardiomyopathies
Source: BMC Cardiovasc Disord. 2026 Oct 22;26:964. doi: 10.1186/s12872-026-06808-3 (PMC13693005; doi:10.1186/s12872-026-06808-3)
Supplement: Supplementary file 1 [file 12872_2026_6808_MOESM1_ESM.docx]

**Supplementary Figures**

**Heart-reactive antibody levels**

**differentially associate with ventricular function**

**in hereditary cardiomyopathies**

Dennis Schiffer^a,b^, Boshra Afshar^c^, Daria Grosser^c^, Juliana Pet^c^, Lena Schmidbauer^e^, Maximilian Bauser^a^, Gustavo Ramos^a^, Valerie Boivin-Jahns^f^, Roland Jahns^d^, Stefan Frantz^a,b^, Peter Heuschmann^e^, Stefan Störk^a,b^, Brenda Gerull^b,g*^, Niklas Beyersdorf^c*^, Caroline Morbach^a,b*^

^a^ *University Hospital Würzburg, Department of Clinical Research & Epidemiology, Comprehensive Heart Failure Center, Germany*

*^b^ University Hospital Würzburg, Department of Medicine I, Germany*

*^c^ University of Würzburg, Institute for Virology and Immunobiology, Germany*

*^d^ University Hospital Würzburg, Interdisciplinary Bank of Data and Biomaterials (ibdw), Germany*

*^e^ University of Würzburg, Institute for Clinical Epidemiology and Biometry, Germany*

*^f^ University of Würzburg, Institute for Pharmacology and Toxicology, Germany*

*^g^ University Hospital Würzburg, Department of Cardiovascular Genetics, Comprehensive Heart Failure Center (CHFC), Germany*

*equal contribution

**Corresponding author:**

PD Dr. med. Niklas Beyersdorf

Institute for Virology and Immunobiology

Versbacher Str. 7

97078 Würzburg

Phone: +49 931-31-81527

Mail: niklas.beyersdorf@vim.uni-wuerzburg.de

**Supplementary Figure Legends**

**Supplementary Fig. S1 Differences in NT-proBNP and NYHA class by HRA levels**

**(a)** Correlation analyses between NT-proBNP concentrations and antibody binding levels for all investigated cardiac antigens (β1AR-EC_II_, TPI3, TPM1, MYH6, MYH7, and MYL7). Results are presented in tabular format, including correlation coefficients and corresponding p-values. No significant associations were observed. **(b)** Association of antibody binding levels with NYHA functional class. Data are presented as comparative analyses across NYHA I–III, with p-values shown in the corresponding table. Among all investigated antigens, only anti-TPM1 antibody levels showed a statistically significant difference across NYHA classes, with elevated TPM1 binding observed in patients with NYHA class II compared to those with NYHA class I or III. Spearman's Rho-correlation: *p<0.05. Number of observations: n=100 for each graph.

**Supplementary Fig. S2 Differences in HRA levels according to HF-subtype**

Comparison of antibody binding levels between patients with heart failure with preserved ejection fraction (HFpEF), mildly reduced ejection fraction (HFmrEF), and reduced ejection fraction (HFrEF). Antibody reactivities against the investigated cardiac antigens are presented stratified by heart failure phenotype. Among the investigated antibodies, anti-MYH6 antibody binding levels were significantly higher in patients with HFrEF compared to those with HFpEF. No consistent differences were observed for the other antibody specificities between heart failure subgroups. Mann-Whitney-U-test: *p<0.05. Number of observations: n=100.

**Supplementary Fig. S3 Differences in gCMP antibody levels according to affected cellular substructure**

Comparison of antibody binding levels stratified by the underlying genetic substrate of cardiomyopathy (sarcomeric, desmosomal, or other gene categories). Antibody reactivities against the investigated cardiac antigens are presented according to the respective affected cellular substructure. Among all antibody specificities, anti-MYH6 antibody levels were significantly higher in patients with variants in non-sarcomeric and non-desmosomal (“other”) genes compared to those with sarcomeric or desmosomal variants. No consistent differences were observed for the remaining antibody specificities across genetic subgroups.

**Supplementary Fig. S4 Genetic variants identified in the gCMP cohort**

The table summarizes the 15 different genetic variants identified in the gCMP cohort, with the corresponding Online Mendelian Inheritance in Man (OMIM) numbers shown on the left. The middle column displays the frequency of each genetic variant across the four cardiomyopathy subtypes, arrhythmogenic right ventricular cardiomyopathy (ARVC), dilated cardiomyopathy (DCM), hypertrophic cardiomyopathy (HCM), and restrictive cardiomyopathy (RCM), respectively. Frequencies are presented as absolute numbers in the format ARVC/DCM/HCM/RCM. On the right, genetic variations were classified as either pathogenic or likely pathogenic (P/LP) or variant of unknown significance (VUS). Their absolute frequencies are reported. Overall, the frequency of P/LP variants was 83/100, while the frequency of VUS was 17/100. In individual cases, two variants were identified per gCMP.

**Supplementary Fig. S5 HRA Binding levels according to genetic variant**

HRA binding levels are shown for the 15 different genetic variants identified in the gCMP cohort. Respective antigens are displayed on the x-axis, while antibody binding levels are shown on the y-axis. For each genetic variant, individual bars represent the binding levels of the six analyzed cardiac autoantibodies distinguished by color. Error bars represent the standard error of the mean.

**Supplementary Fig. S6 Proteins included in the bead assay**

The table summarizes the six proteins and peptides targets analyzed in the bead-based assay.

**Supplementary Fig. S7 Antibodies included in the bead assay**

The table summarizes the antibodies analyzed using the bead-based assay, including their dilutions.

**Differences in NT-proBNP and NYHA class by HRA levels**

**Supplementary Fig. S1**

**Differences in HRA levels according to HF-subtype**

**Supplementary Fig. S2**

**gCMP**

**Supplementary Fig. S3**

**Prevalence of cellular substructural component**

**Differences in gCMP antibody levels according to affected cellular substructure**

56%

21%

22%

21%

56%

21%

22%

**Cellular substructure**

**Genetic variants identified in the gCMP cohort**

**Supplementary Fig. S4**

| **Genetic variant (OMIM identifier)** | **Associated gCMP: ARVC/DCM/HCM/RCM (n)** | **Classification (n)** |
| --- | --- | --- |
| PKP2 (602861) | 12/0/0/0 = 12 | P/LP = 12, VUS = 0 |
| DSP (125647) | 5/1/0/1 = 7 | P/LP = 5, VUS = 2 |
| TTN (188840) | 0/8/2/0 = 10 | P/LP = 8, VUS = 1 |
| MYH7 (160760) | 0/6/13/0 = 19 | P/LP = 14, VUS = 5 |
| FLNC (102565) | 1/6/3/2 = 12 | P/LP = 9, VUS = 3 |
| LMNA (150330) | 0/2/0/0 = 2 | P/LP = 2, VUS = 0 |
| TNNC1 (191040) | 0/1/0/0 = 1 | P/LP = 0, VUS = 1 |
| TNNT2 (191045) | 0/1/2/0 = 3 | P/LP = 1, VUS = 2 |
| MYBPC3 (600958) | 0/0/18/0 = 18 | P/LP = 18, VUS = 0 |
| TNNI3 (191044) | 0/0/6/0 = 6 | P/LP = 6, VUS = 0 |
| TPM1 (191010) | 0/0/1/0 = 1 | P/LP = 1, VUS = 0 |
| ACTC1 (102540) | 0/0/1/0 = 1 | P/LP = 0, VUS = 1 |
| DSG2 (125671) | 2/0/0/0 = 2 | P/LP = 2, VUS = 0 |
| LMNA (150330) | 0/6/0/0 = 6 | P/LP = 6, VUS = 0 |
| MYL3 (160790) | 0/0/1/0 = 1 | P/LP = 1, VUS = 0 |

**HRA Binding levels according to genetic variant**

**Supplementary Fig. S5**

**Proteins included in the bead assay**

 **Proteins and peptides:**

**Supplementary Fig. S6**

**Antibodies included in the bead assay**

**Primary and secondary Antibodies:**

**Supplementary Fig. S7**
